# A Difference-In-Differences Study of the Effects of a New Abandoned Building Remediation Strategy on Safety

**DOI:** 10.1371/journal.pone.0129582

**Published:** 2015-07-08

**Authors:** Michelle C. Kondo, Danya Keene, Bernadette C. Hohl, John M. MacDonald, Charles C. Branas

**Affiliations:** 1 United States Department of Agriculture-Forest Service, Northern Research Station, Philadelphia, PA, United States of America; 2 Department of Biostatistics and Epidemiology, Perelman School of Medicine, University of Pennsylvania, Philadelphia, PA, United States of America; 3 Social and Behavioral Sciences, Yale School of Public Health, New Haven, CT, United States of America; 4 Department of Criminology, School of Arts & Sciences, University of Pennsylvania, Philadelphia, PA, United States of America; Peking UIniversity, CHINA

## Abstract

Vacant and abandoned buildings pose significant challenges to the health and safety of communities. In 2011 the City of Philadelphia began enforcing a Doors and Windows Ordinance that required property owners of abandoned buildings to install working doors and windows in all structural openings or face significant fines. We tested the effects of the new ordinance on the occurrence of crime surrounding abandoned buildings from January 2011 to April 2013 using a difference-in-differences approach. We used Poisson regression models to compare differences in pre- and post-treatment measures of crime for buildings that were remediated as a result of the ordinance (n = 676) or permitted for renovation (n = 241), and randomly-matched control buildings that were not remediated (n = 676) or permitted for renovation (n = 964), while also controlling for sociodemographic and other confounders measured around each building. Building remediations were significantly associated with citywide reductions in overall crimes, total assaults, gun assaults and nuisance crimes (*p* <0.001). Building remediations were also significantly associated with reductions in violent gun crimes in one city section (*p* <0.01). At the same time, some significant increases were seen in narcotics sales and possession and property crimes around remediated buildings (*p* <0.001). Building renovation permits were significantly associated with reductions in all crime classifications across multiple city sections (*p* <0.001). We found no significant spatial displacement effects. Doors and windows remediation offers a relatively low-cost method of reducing certain crimes in and around abandoned buildings. Cities with an abundance of decaying and abandoned housing stock might consider some form of this structural change to their built environments as one strategy to enhance public safety.

## Introduction

Housing abandonment and vacancy is increasing in the US [1]. This is especially the case in the centers of so-called “legacy” cities where, over the past half century, processes such as suburbanization, residential flight, urban disinvestment, and redlining have resulted in rapidly increasing numbers of vacant properties [2]. Post-industrial cities like Chicago, Cleveland, Detroit, Pittsburgh, Philadelphia and Baltimore have also experienced rapid declines in manufacturing jobs further exacerbating residential flight and the growth of vacant properties. As one example, in 2010 Philadelphia had some 40,000 vacant properties, including over 3,000 with abandoned buildings or other structures [3].

Vacant and abandoned properties have a significant economic impact on property owners [4]. Vacant properties in Philadelphia have been shown to reduce property values city-wide by 6.5% (an average of $8,100) and by up to 20% in some neighborhoods, representing $3.6 billion in total lost wealth for the city [3]. Loss in property value was as high as 20% in some city neighborhoods [3]. Vacant properties also represent a substantial economic burden on municipalities themselves, in the form of lost tax revenues and costs associated with maintenance, remediation, and policing [1]. Vacant properties cost the city of Philadelphia over $20 million per year in maintenance and an additional $2 million per year in uncollected property taxes [3].

Research suggests that vacant and abandoned properties also have a negative impact on communities’ health and safety. For example, research in Philadelphia found an association between the presence of neighborhood vacant properties and an increased risk of neighborhood assaults [5]. Beyond Philadelphia, other studies have found associations between presence of boarded-up buildings with drug-dependence mortality [6], rates of sexually-transmitted disease [7], and premature mortality [8]. Moreover, residents in neighborhoods with numerous abandoned homes and vacant lots describe the many negative impacts these features have on community well-being, physical, and mental health [9]. According to these residents, vacant properties reduce community cohesion, invite trash, rodents, and crime and increase fear, stress and anxiety [9].

A number of potential mechanisms explain why abandoned properties generate negative crime and health outcomes. Broken windows theory [10], for example, posits that visible disorders such as vacant buildings and lots signify that a neighborhood is uncared-for, has little informal surveillance by those who live there, and that various incivilities are tolerated. These perceptions of disorder are thought to send a signal to would-be offenders that committing crime is acceptable, and likely to go unchallenged or unseen. A substantial body of research supports the relationship between perceived incivilities and other subjective measures such as neighborhood satisfaction and fear of crime [11]. Others have suggested that structural conditions such as poverty, rather than physical disorder, are the primary sources of crime and fear of crime [12, 13]. Higher concentrations of poverty are thought to erode a neighborhood’s collective efficacy, or the ability to engage in shared expectations of social control related to neighborhood problems and neighborly trust, which in turn is correlated with crime [13]. Criminological research, often taking a social-ecological approach, has examined the relationships between neighborhood disadvantage, physical disorder and crime [14–17]. These studies have not used experimental or quasi-experimental approaches to test the effects of how varying environmental attributes of places affects crime. As an exception, one study which included six controlled field experiments found that presence of physical disorder (such as presence of graffiti) led to a significantly higher occurrence of minor offenses (such as littering) [18].

While researchers continue to disentangle the relationships between poverty, physical disorder and crime, cities have been forced into action, initiating highly innovative but largely untested programs that attempt to abate physical signs of disorder and revitalize their neighborhoods, reduce expenses, generate tax revenues, and potentially improve public safety [19–21]. These programs involve, for example, demolishing vacant buildings or cleaning and greening vacant lots. Some of these programs have shown promising results in terms of effects on health and crime. For example, a study of vacant-lot greening programs in Philadelphia found that greening was associated with reduced gun assaults and vandalism, as well as improved health outcomes such as less stress and more exercise in one section of the city [22]. Other programs, like the movement to demolish abandoned buildings, could alleviate the problem of vacant housing but might also potentially have unintended effects associated with the creation of vacant properties overall [23].

Municipalities are searching for effective programs to deal with problems associated with vacant properties [1]. Cities such as Philadelphia and Chicago have recently begun implementing more creative vacant property remediation strategies. More specifically, Philadelphia enacted a Doors and Windows Ordinance in 2011 to require abandoned-building owners to install working doors and windows in all structural openings. Section 306 of the Philadelphia Property Maintenance Code [24] states that “Where such doors or windows or entrance to openings are readily accessible to trespassers, they shall be kept securely locked, fastened or otherwise secured. The owner shall take any other measures prescribed by the Department to prevent unauthorized entry to the premises by closing all openings with materials approved by the Department.” In line with a broken windows theory, this remediation of abandoned buildings may lessen the sense and appearance of physical disorder, thereby reducing the incidence of crime in an area. Securing buildings in this way may also alter physical opportunities for crime–replacing plywood boards with functional doors and windows that prohibit easy entry and are see-through may help reduce squatting, drug dens, and other crimes that proliferate when they are concealed from sight in abandoned buildings. However, there have been no published quasi-experimental studies that test the effect of this form of blight reduction on subsequent neighborhood crime. We addressed this gap in knowledge by conducting a difference-in-differences analysis of the effect of Philadelphia’s Doors and Windows Ordinance on surrounding crime levels.

## Materials and Methods

In 2010, Philadelphia identified approximately 25,000 vacant buildings, and enacted legal tools to hold owners responsible for property remediation. The main tool was the Doors and Windows Ordinance, which was signed into law in January 2011. This ordinance allows the city to fine owners for a building opening that is not covered with a functional door or window on blocks that are more than 80% occupied. Plywood coverings deteriorate quickly, look disheveled, signal obvious blight, and are often penetrated to allow illegal entry into abandoned buildings. Under this ordinance, plywood is deemed an unacceptable door and window covering [24].

**Fig 1:**
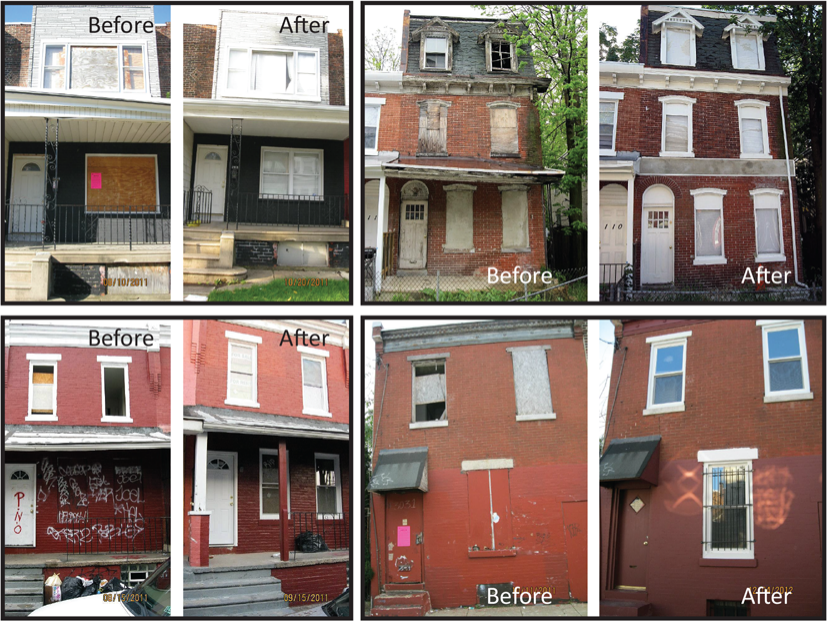
Before and After Photos of Renovated Vacant Properties. This figure shows four properties that received doors-and-windows remediation. Pink posters on doors of properties shown in the upper left- and lower right-hand quadrants notify the owner of a date by which the structure must be in compliance or face penalty.

The city began issuing Out of Compliance citations in January 2011. This involves posting a notice on the abandoned structure (pink notices shown in Fig 1) and sending a notice of citation to the identified owner indicating that they will be taken to court if they fail to comply with the ordinance. The city records the number of openings out of compliance, and assesses fines of $300 per day per opening. As of May 2014, the Licensing and Inspection Department had cited 2,356 buildings, and assessed at least $1.5 million in fines. To make it easier for property owners to comply in a timely manner, the city does not require them to obtain renovation permits to meet the ordinance requirements. Inspectors visit the property approximately every 35 days to assess compliance.

Owners intending to renovate their properties above and beyond door and window replacements are required to apply for a renovation permit. Although there is no record of whether owners actually did renovations, filing for a permit does not exempt someone from compliance with the ordinance and officials from the Licensing and Inspection Department have suggested that renovations “almost always occur” after permits are filed. Thus, we interpret the filing of a renovation permit as remediation that goes beyond compliance with the ordinance.

**Fig 2:**
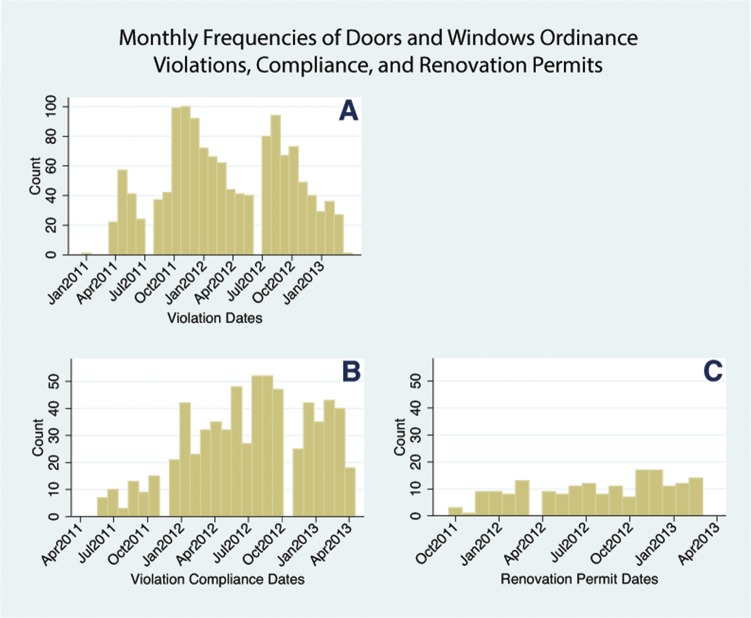
Monthly Frequencies of Violation Citations (A), Violation Compliances (B) and Renovation Permits (C) between January 2011 and May 2013.

One market analysis found that areas with clustered compliance properties (“Neighborhood Enforcement Clusters”) had an average increase in home sales price of 32%, compared to a 2% increase at control locations between 2008 and 2012 [25, 26]. Compliance clusters also had a lower rate of tax delinquency than control properties [26]. We examined the effect of both violation compliance and renovation permits on crime counts surrounding abandoned buildings. We used all compliance locations (N = 676) and renovation permits (N = 241) as treatment sites. Fig 2 shows the range and frequency of dates for each of these permit status conditions. As shown in sub-graph A, the city issued up to 100 violations per month. Sub-graphs B and C show that up to approximately 50 properties complied with the ordinance per month. The monthly frequency of renovation permits issued was in general lower than violation compliances.

We compared crime occurrence at buildings that complied with the ordinance (treatment sites or buildings that have been secured or abated according to the ordinance) to buildings that remained un-renovated (i.e. wait-list, matched control sites) and had received citations. We matched treatment with control sites using a randomization method in which we assigned random numbers to available treatment and control sites, and matched these sites in order based on the numbering system. After initial matching, we manually removed and replaced any control lot within 1/4-mile of its matched treatment lot to avoid treatment spill-over and contamination, as well as artificial dilution of effect due to immediate proximity.

**Fig 3:**
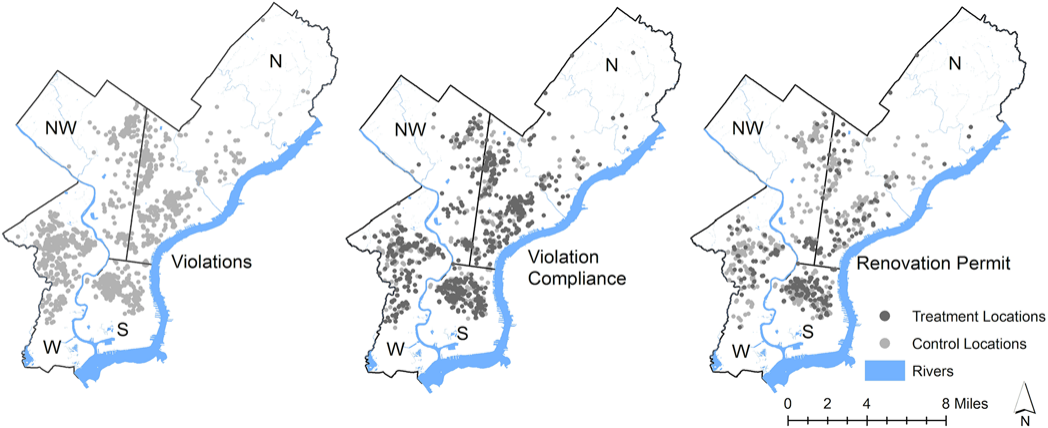
Map of Doors and Windows Ordinance violations, and treatment and control locations for violation compliance and renovation permits in Philadelphia, PA. Data source: Philadelphia Department of Licensing and Inspection (2013) North (N), Northwest (NW), South (S) and West (W) sections are indicated.

We conducted this random matching process within each of four city sections (see Fig 3) in order to control for confounding variables relating to geography or section location. Sections are well-known within Philadelphia because they are separated by significant boundaries, such as rivers, parks, or major thoroughfares. Each section has its own characteristics, some of which may confound the effect of housing changes on crime patterns. These areas also tend to have a higher percent of population living in poverty. Fig 3 also shows treatment site locations and violation compliance treatment sites were an average of 608 feet apart and renovation permit treatment sites were an average of 1,026 feet apart. The difference-in-differences modeling approach can generate an unbiased estimate of the effect of the ordinance on changes in crime for those willing to comply with the intervention, even if conditions at control and treatment sites are not comparable [27].

For the Violation Compliance outcome, we used a 1:1 treatment to control ratio (N = 676). For the Renovation Permit outcome, we used a 1:4 treatment site (N = 241) to control site (N = 964) ratio. These ratios were determined given available sample size and proximity criteria. Within matched groups, we assigned treatment dates to controls so that each comparison would be followed for the same period of time. For each outcome, we examined treatment and control groups as a balanced time series.

We assessed outcomes from the start of July 2010 to the end of July 2013 to allow for at least 10 months pre-treatment and 3 months post-treatment at all treatment sites. We assessed effects of violation compliance occurrences between June 2011 and April 2013. The mean pre-treatment period for violation compliances was 25 months (SD = 5.5, range = 11.4 to 33.7 months) and the mean post-treatment period was 12 months (SD = 5.5, range = 2.9 to 25.2 months). For renovation permits the mean pre-treatment period was 26 months (SD = 5, range = 14.3 to 33.8 months), and post-treatment period mean was 11 months (SE = 5, range = 2.8 to 22.3 months).

### Data

The Philadelphia Department of Licenses and Inspections provided data on 2,356 violations, including address, location (in latitude-longitude coordinates), date of violation, number of openings, date of violation compliance and date of renovation permit. The Philadelphia Police Department provided dates and latitude-longitude coordinates for 14 classes of crimes occurring between 2010 and 2013. We tested treatment effects on the following crime classifications: 1) all crimes, 2) violent gun crimes (gun assaults and gun robberies), 3) gun assaults, 4) all assaults, 5) all robberies, 6) property crimes (burglaries and thefts), 7) narcotics sales and possession, 8) nuisance crimes type I (vandalism and illegal dumping), and 9) all nuisance crimes (public drunkenness, disorderly conduct, vandalism and illegal dumping). We include measures of the counts of crime around each building location by month.

We obtained demographic information at the Census block group level from the U.S. Census Bureau. We assigned estimates from the American Community Survey to years 2010–2013. We incorporated the following demographic indicators that could confound the relationship between crime and the doors-and-windows building treatment: 1) median age of the population; 2) percent of the population age 25 and older with less than a high school-level education; 3) percent of the population living below 100% of the federal poverty level; and 4) median annual household income. While multiple studies have found race or ethnicity to be associated with neighborhood distress, we found that it correlated with our other demographic indicators, and it did not alter findings from statistical models described below.

We calculated demographic measures and crime outcomes in the areas surrounding each building location for each month of the study using ArcGIS (v10.1, ESRI, Inc., Redlands, CA). We calculated kernel density and Census tract-level counts at each study location. The kernel density method creates a smooth surface using a quadratic function from a set of points, in this case crime locations. Each location on the new grid surface is assigned a density value, or magnitude per unit area. This method more accurately measures the magnitude of effect for point-based outcomes such as crime; the effects of a crime will be felt not just at the exact location of occurrence but also in surrounding areas. For point-based kernel density calculations, we created 100-foot resolution grids representing kernel density of crimes based on a one-mile search radius. For Census-tract calculations, we linked crime counts to census tract centroids and used these to create inverse-distance weighted (IDW) measures at each site (using a cell size of 100 feet and search radius of 12 points). The IDW method also creates a smooth surface by interpolating values assigned to points. For demographic measures we used the same process but generated IDW measures at the block group level.

### Statistical Analyses

We first conducted unadjusted analyses using summary statistics, cross-tabulations, and tests for normality and multicollinearity. Multicollinearity was minimal (all variance-inflation factors < 3.0). We used Poisson random-effects regression models to estimate the impact of violation compliance and renovation permit on crime outcomes (Y), while controlling for demographic variables (negative binomial models were first attempted but would not converge) [28]. We estimated parameters with Eq 1 below:

$$Y_{it}=\beta_{0}+\beta_{1}P_{it}+\beta_{2}R_{it}+\beta_{1}(P_{it}\times R_{it})+\beta_{4}t+\beta_{5}O_{i}+\beta_{6}M_{i}+\sum_{k=3}^{p}\beta_{k}S_{i}+\sum_{k=4}^{p}\beta_{k}X_{it}+\xi_{i}+\varepsilon_{it}$$ (1)

The units of observation were violation compliance or renovation permit (i) per month of the study (t). The variable of interest was a difference-in-differences term, *P*_*it*_ × *R*_*it*_, with *P*_*it*_ indicating pre-compliance (0) / post–compliance (1) status (or pre-permit and post-permit status), and *R*_*it*_ indicating treatment (1) /control (0) site status. The *β*_3_ interaction coefficient of the difference-in-differences term estimates the effect of the treatment on the outcome [29].

Each regression model (see Eq 1) included a crime outcome *Y*_*it*_; a pre-post compliance term *β*_1_ *P*_*it*_; a treatment-control status term *β*_2_ *R*_*it*_; a difference-in-differences term *β*_3_(*P*_*it*_ × *R*_*it*_); a term indicating time, *β*_4_ *t*; a term *β*_5_ *O*_*i*_ indicating the number of window or door openings on each vacant building; a pre-period mean outcome interaction term to adjust for regression to the mean *β*_6_ *M*_*i*_, terms indicating section of the city, *S*_*i*_; and a series of *p* independent demographic covariates, *X*_*it*_; and residual error, *ε*_*it*_. For section-specific models, we included a random-effects (building specific) parameter for each violation compliance or renovation permit, *ξ*_*i*_; and we allowed residual error *ε*_*it*_ to vary by location using robust standard errors [30]. We restricted demographics covariates to pre-treatment levels. We estimated models for point-based and Census-tract based crime counts, and compared estimates by city section.

*P*-values less than 0.01 indicated significant effect. We used this lower *p*-value to account for multiple testing issues, i.e. by chance alone 5 out of 100 tests (5%) could be statistically significant. We calculated Incidence Rate Ratio; or the ratio of crime counts per square mile at the treatment site to that of the control site. In addition, we calculated the expected change in the number of crimes around the treatment sites due to remediation.

### Test for Displacement

In order to test for spillover effect, or displacement of crimes, we calculated crime counts at three contiguous radii expanding from each project site: 1) within one-eighth mile radius from each site, 2) between one-eighth and one-quarter miles from each site, and 3) between one-fourth and one-half miles from each site. We calculated counts of crimes with significant difference-in-differences effects found in regression models described above. Treatment sites were those that met the violation compliance outcome only. We used an aggregated time step of three months instead of every month to reduce the influence of random monthly fluctuations on our estimates, for a total of 12 three-month time periods. We used the same model described above to derive difference-in-differences estimates at each geographic level, and compared estimates at the three geographic levels surrounding each project site. A decrease in crimes immediately surrounding project sites, but increase in crimes in surrounding areas, would indicate a displacement or spatial pushing-effect on crimes.

## Results

Between January 2011 and April 2013, 29% (676 out of 2,356 cited buildings) complied with the Doors and Windows ordinance. For the Violation Compliance outcome, control sites were not statistically different from treatment sites in terms median age, median household income, percent of the population with less than a high school-level education and percent of households earning less than the federal poverty standard. For the Renovation Permit outcome, control sites were not statistically different from treatment sites in terms median age. However, control sites were different at various levels in terms of median income (control sites lower than treatment sites), percent of households earning less than the federal poverty standard (control sites higher than treatment sites), and percent of the population with less than a high school-level education (control sites higher than treatment sites; *p*<0.01). All sociodemographic variables served as covariates in our final regression models to control for these group differences.

Tables 1 and 2 show difference-in-differences coefficient estimates, represented as Incidence Rate Ratio (IRR) of crime outcomes associated with violation compliance from the random-effects Poisson models. Table 1 shows results from point-based models of the effects of violation compliance on crime overall and by different classifications for the city as a whole and divided by the four sections. City-wide, with the exception of violent gun crimes, narcotics sales and possession, and vandalism and illegal dumping, ordinance compliance was associated with significant decreases in all crime categories. The size and significance of these effects, however, varied by section of the city. Over a one-year period in areas around buildings that had been remediated as a result of violation compliance versus not, there were an estimated 8 fewer assaults, 10 fewer gun assaults and 5 fewer nuisance crimes.

**Table 1:** Adjusted Difference-in-Differences Estimates of Violation Compliance on Point-Level Crime Outcomes, by City Section, Philadelphia, PA, January 2010 –April 2013^1^.

	All Philadelphia			Northwest			North			South			West		
	IRR	SE		IRR	SE		IRR	SE		IRR	SE		IRR	SE	
**All crimes**	0.99	0.00	**	0.96	0.02		1.01	0.01		0.99	0.01		0.97	0.01	**
Violent gun crimes	1.00	0.01		1.02	0.07		1.03	0.04		0.95	0.04		0.96	0.03	
**All assaults**	0.98	0.00	***	0.95	0.03		0.99	0.02		0.97	0.02		0.99	0.02	
**Gun assaults**	0.96	0.01	***	0.94	0.06		0.98	0.03		0.92	0.04	*	0.97	0.07	
**Robberies**	1.01	0.00	**	1.01	0.04		1.03	0.02		1.01	0.03		0.97	0.02	
**Narcotics sales & possession**	1.03	0.00	***	0.90	0.04	*	1.06	0.01	***	0.96	0.02		0.94	0.04	
**Property crimes**	1.03	0.00	***	0.90	0.04	*	1.06	0.09		0.96	0.02		0.94	0.04	
Vandalism & illegal dumping	0.99	0.00		0.93	0.03	*	1.00	0.01		1.00	0.01		1.00	0.01	
**All nuisance crimes**	0.99	0.00	***	0.96	0.02		0.99	0.01		0.99	0.01		0.98	0.01	*

* p<0.05

**p<0.01

***p<0.001

1. All estimates include controls for median age, median household income, percent of the population with less than a high school-level education, and percent of households earning less than the federal poverty standard. 2. IRR: Incidence Rate Ratio; ratio of incidence rate of crimes per square mile at the treatment site to incidence rate of crimes per square mile at the control site 3. SE: Standard Error

**Table 2:** Adjusted Difference-in-Differences Estimates of Violation Compliance on Census-Tract Level Crime Outcomes, by City Section, Philadelphia, PA, January 2010 –April 2013^1^.

	All Philadelphia			Northwest			North			South			West		
	IRR	SE		IRR	SE		IRR	SE		IRR	SE		IRR	SE	
**All crimes**	0.99	0.00		0.97	0.01	*	1.00	0.01		0.97	0.01	***	-	-	
**Violent gun crimes**	1.00	0.01		0.97	0.08		1.03	0.02		0.83	0.05	**	1.04	0.04	
All assaults	0.98	0.01	*	0.87	0.08		0.99	0.03		0.81	0.08	*	1.08	0.05	
Gun assaults	0.98	0.01		0.92	0.05		1.00	0.02		0.92	0.04	*	1.03	0.03	
Robberies	1.00	0.02		0.94	0.06		1.00	0.03		0.95	0.04		1.02	0.03	
**Narcotics sales & possession**	1.05	0.05		0.94	0.07		1.07	0.01	***	0.92	0.04		0.93	0.1	
**Property crimes**	1.05	0.01	***	0.94	0.07		1.07	0.01	***	0.92	0.04	*	0.93	0.06	
Vandalism & illegal dumping	1.01	0.01		0.99	0.04		1.02	0.02		1.01	0.02		1.00	0.02	
All nuisance crimes	1.00	0.01		0.98	0.03		1.00	0.01		1.01	0.02		-	-	

* p<0.05

**p<0.01

***p<0.001

1. All estimates include controls for median age, median household income, percent of the population with less than a high school-level education, and percent of households earning less than the federal poverty standard.2. IRR: Incidence Rate Ratio; ratio of incidence rate of crimes per square mile at the treatment site to incidence rate of crimes per square mile at the control site 3. SE: Standard Error.

4. “-”indicates that numbers are too small to report

In general, estimates from tract-based models shown in Table 2 were smaller and not statistically significant. Compliance with the ordinance was associated with a statistically significant reduction in total crimes in South Philadelphia (12 fewer per year). In addition, both point- and tract-based models reported statistically significant increases in narcotics sales and possession and property crimes (city-wide and in North Philadelphia) around buildings remediated as a result of violation compliance versus not.

Filing dates indicate that approximately 10% (n = 241) of cited buildings requested a renovation permit. One hundred and nine of these buildings had also complied with the doors and windows ordinance, while the rest were still out of compliance in July 2013. Regression-adjusted estimates from Poisson models of renovation permits on crime outcomes are shown in Tables 3 and 4. The estimated effects for renovation permits on crime were on average larger than those shown for compliance with the city ordinance. Filing for renovation permits was associated with significant reductions city-wide (*p*<0.001) for all crime categories in point-based models. Across at least three different city sections there were significant reductions in total crimes, violent gun crimes, narcotics sales and possession, and property crimes around buildings that filed for renovation permits. The largest statistically significant decrease in terms of number of crimes occurring around renovation permitted-buildings was in gun assaults (6 fewer city-wide, and 5 fewer in South Philadelphia in a one-year period). No statistically significant increases in crimes occurred around renovation-permitted buildings.

**Table 3:** Adjusted Difference-in-Differences Estimates of Renovation Permit on Point-Level Crime Outcomes, by City Section, Philadelphia, PA, January 2010 –April 2013^1^.

	All Philadelphia			Northwest			North			South			West		
	IRR	SE		IRR	SE		IRR	SE		IRR	SE		IRR	SE	
**All crimes**	0.96	0.00	***	0.91	0.02	***	0.95	0.03		0.99	0.00	*	0.95	0.01	***
**Violent gun crimes**	0.90	0.01	***	0.81	0.06	**	1.00	0.07		0.87	0.03	***	0.95	0.04	
**All assaults**	0.92	0.00	***	0.93	0.03	*	0.92	0.03	*	0.92	0.02	***	0.97	0.04	
**Gun assaults**	-	-		0.91	0.05		0.86	0.05	*	0.82	0.04	***	-	-	
**Robberies**	0.98	0.01	***	0.92	0.05		1.05	0.04		1.01	0.03		0.94	0.03	
**Narcotics sales & possession**	0.87	0.01	***	0.78	0.05	***	0.94	0.01	***	0.97	0.02		0.84	0.05	**
**Property crimes**	0.87	0.01	***	0.77	0.05	***	0.94	0.08		0.96	0.02		0.85	0.05	**
**Vandalism & illegal dumping**	0.98	0.01	***	0.88	0.03	***	0.96	0.02		1.00	0.01		0.99	0.02	
**All nuisance crimes**	0.98	0.00	***	0.94	0.02	***	0.97	0.02		-	-		0.97	0.01	*

* p<0.05

**p<0.01

***p<0.001

1. All estimates include controls for median age, median household income, percent of the population with less than a high school-level education, and percent of households earning less than the federal poverty standard. 2. IRR: Incidence Rate Ratio; ratio of incidence rate of crimes per square mile at the treatment site to incidence rate of crimes per square mile at the control site 3. SE: Standard Error.

4. “-”indicates that numbers are too small to report

**Table 4:** Adjusted Difference-in-Differences Estimates of Renovation Permit on Census-Tract Level Crime Outcomes, by City Section, Philadelphia, PA, January 2010 –April 2013^1^.

	All Philadelphia			Northwest			North			South			West		
	IRR	SE		IRR	SE		IRR	SE		IRR	SE		IRR	SE	
**All crimes**	0.94	0.00	***	0.85	0.03	***	0.97	0.04		0.97	0.01	*	0.96	0.02	
**Violent gun crimes**	0.87	0.02	***	0.83	0.10		1.01	0.12		0.81	0.06	***	0.96	0.05	
**All assaults**	0.92	0.01	***	1.03	0.07		0.94	0.04		0.88	0.03	***	1.00	0.05	
**Gun assaults**	0.86	0.03	***	1.08	0.10		0.88	0.06	*	0.71	0.06	***	-	-	
**Robberies**	0.96	0.01	**	0.95	0.08		1.04	0.06		0.98	0.03		0.95	0.05	
**Narcotics sales & possession**	0.89	0.01	***	0.78	0.07	**	1.04	0.03		0.98	0.04		0.82	0.07	*
**Property crimes**	0.88	0.01	***	0.78	0.08	**	1.03	0.11		0.97	0.04		0.82	0.07	*
**Vandalism & illegal dumping**	0.97	0.01	**	0.83	0.04	***	0.97	0.03		0.98	0.02		1.02	0.03	
**All nuisance crimes**	0.95	0.01	***	0.88	0.03	***	0.94	0.04		0.97	0.02		0.99	0.03	

* p<0.05

**p<0.01

***p<0.001

1.All estimates include controls for median age, median household income, percent of the population with less than a high school-level education, and percent of households earning less than the federal poverty standard. 2. IRR: Incidence Rate Ratio; ratio of incidence rate of crimes per square mile at the treatment site to incidence rate of crimes per square mile at the control site 3. SE: Standard Error

4. “-”indicates that numbers are too small to report.

Tests for spillover effect found a reducing effect size with increasing distance away from buildings after treatment. Within one-eighth mile of building treatment sites, models showed a non-significant 1% decrease in total crimes. Between one-eighth and one-quarter miles from building treatment sites, there was a non-significant 1% decrease and between one-quarter and one-half miles from building treatment sites there was a 1% decrease in total crimes.

## Discussion

Vacant and abandoned buildings pose significant challenges to the health and safety of communities. This study is the first to demonstrate the effects of abandoned building remediation on changes in surrounding crime. Renovation permits consistently were associated with crime declines around building sites. Compliance with the city ordinance to install a functional door or windows on vacant and abandoned buildings had smaller effects on surrounding crime compared to those buildings where owners filed for renovation permits. City-wide, we found significant reductions in total crimes, assaults, gun assaults, robberies and nuisance crimes associated with ordinance compliance, using both Census-tract and point-based estimates for buildings that were compliant with the ordinance. At the same time, tract-based models showed increases in narcotics sales and possession and property crimes (city-wide and in North Philadelphia) at buildings remediated as a result of violation compliance versus not, depending on city section.

The most statistically significant reductions around ordinance-compliance properties were seen in serious “Part 1” crimes [31] (total assaults and gun assaults) as well as nuisance crimes. While regression models showed some significant effects within city sections, our models are not able to reveal external influences on (for example concentrated policing efforts in this area that might affect crime occurrence rate) or mechanisms of this geographically-specific association.

We also did not find strong evidence of a spillover effect on crime outcomes. Regression-adjusted estimates of total crimes showed declining effect size with increasing distance from building treatment locations. These effects were non-significant and therefore we should not interpret a spillover benefit, but rather as no clear evidence of crime displacement.

Abandoned, boarded-up buildings are one aspect of neighborhood decay that may contribute to crime and therefore serve as a potential intervention for crime reduction. Several prior studies have found associations between vacant buildings and increases in crime and neighborhood violence [32–34]. However, to our knowledge, our study is the first to examine the impact of property abatement changes on crime using a quasi-experimental approach. Our study shows that remediating abandoned buildings reduces total crimes and many forms of violence and nuisance crimes.

The significant reductions that we found are supported by existing theory, such as the broken windows theory which holds that dilapidated buildings signal social disorder and a tolerance for criminal acts. New doors and windows and a newly cleaned building facade likely signaled to potential offenders that a property was occupied, and therefore crimes in general, and violent and nuisance crimes were not tolerated. A decrease in all classifications of crime around permit renovation properties could also be associated with activities that would no longer be supported by a secured, as opposed to un-secured, vacant building. In this way, the dual-challenge to potential offenders of being seen more easily from the outside through glass windows (as opposed to plywood coverings) *and* entering the openings of abandoned buildings through glass windows that make noise and leave an obvious, lasting visual sign of forced entry when shattered, may have also served as a physical explanation for our findings. Use of other research methods, such as qualitative and ethnographic studies, as well as perhaps time-lapse photography studies of newly remediated buildings could help to better understand and explain the mechanisms behind our findings.

Although focusing on buildings and not land per se, the findings reported here parallel other research showing that remediating vacant lots through greening reduced gun assaults and other crimes [5, 22]. Together, these studies are accruing higher levels of scientific evidence that basic, low-cost structural changes to urban built environments may have lasting and significant effects on public safety and well-being [35].

### Limitations

Violation compliance is a strong treatment measure because it is based on a physical check at each violation site approximately every 35 days. The renovation permit application outcome is less ideal because it represents an administrative status, and does not necessarily indicate a physical renovation actually occurs (though city officials report that in their experience it “almost always” does). Documentation of the type of renovation, and completion status determined in-person, would improve this treatment measure. In addition, a longer evaluation period could also help assess the longer-term effects of the Doors and Windows Ordinance on crime in surrounding areas.

The violation compliance treatment is tied to abandoned buildings located on blocks that are at least 80% occupied, with owners willing to comply with the ordinance. It may be that the effects exist for only the blocks that are more occupied than not. Therefore, remediating buildings in areas that are totally abandoned may not be effective.

Our modeling approach is able to isolate and detect effect of the intervention on crime at treatment sites compared to control sites [29]. While our models control for background socioeconomic conditions at each site that might influence crime levels, our models do not take into account other potential external influences on crime occurrences, such as geographical variation in policing practices that change over time. It is possible that within each city section compliance with the ordinance may change policing patterns, so the effect we observe may be due to the knock-on effect of the shift in policing and not the change in the actual building structure. In addition, violation compliance and renovation permit treatment sites are densely located, which could cause contamination of effect area and underestimation of effects.

In addition, use of administrative boundaries, such as Census tracts, to estimate effects has its limitations. These boundaries are irregularly shaped, and projects rarely center within boundaries. Therefore, we have also used point-based density estimates that offer a more precise way to quantify density of safety outcomes associated with a project site.

## Conclusions

The City of Philadelphia, like many legacy cities, is dealing with an over-supply of vacant and abandoned buildings. Cities are increasingly investing in abandoned-building remediation strategies to reduce blight and crime, stabilize real estate values, and encourage economic development. For example, over 80 cities including Philadelphia have worked to develop LandBank programs that seek to acquire, hold, manage and develop abandoned properties as a means of neighborhood reinvestment. The Doors and Windows Ordinance is a complement to these strategies. Doors and windows remediation programs offer a lower-cost alternative to demolishing abandoned buildings. Our findings suggest that this lower-cost method can possibly be an effective means of reducing crime. Although randomized trials would be required to establish a clearer causal pathway between doors and windows remediation, crime, and other outcomes, this quasi-experimental study provides useful evidence of the potential effect of abandoned building remediation policies on crime in US legacy cities.
