# Correction: A Difference-In-Differences Study of the Effects of a New Abandoned Building Remediation Strategy on Safety

**DOI:** 10.1371/journal.pone.0136595

**Published:** 2015-08-20

**Authors:** Michelle C. Kondo, Danya Keene, Bernadette C. Hohl, John M. MacDonald, Charles C. Branas

In the Materials and Methods section, there is an error in Equation 1 in the first paragraph of the Statistical Analyses section. The second *β*_*1*_ symbol should read *β*_*3*_. Please view the complete, correct equation here:

$$Y_{it}=\beta_{0}+\beta_{1}P_{it}+\beta_{2}R_{it}+\beta_{3}(P_{it}\times R_{it})+\beta_{4}t+\beta_{5}O_{i}+\beta_{6}M_{i}+\sum_{k=3}^{p}\beta_{k}S_{i}+\sum_{k=4}^{p}\beta_{k}X_{it}+\xi_{i}+\varepsilon_{it}$$
